# Electroencephalography in the Diagnosis of Genetic Generalized Epilepsy Syndromes

**DOI:** 10.3389/fneur.2017.00499

**Published:** 2017-09-25

**Authors:** Udaya Seneviratne, Mark J. Cook, Wendyl Jude D’Souza

**Affiliations:** ^1^Department of Medicine, St. Vincent’s Hospital, The University of Melbourne, Melbourne, VIC, Australia; ^2^Department of Neuroscience, Monash Medical Centre, Melbourne, VIC, Australia

**Keywords:** spike-wave, polyspike, sleep, photoparoxysmal response, myoclonic seizure, absence seizure, tonic-clonic seizure, circadian

## Abstract

Genetic generalized epilepsy (GGE) consists of several syndromes diagnosed and classified on the basis of clinical features and electroencephalographic (EEG) abnormalities. The main EEG feature of GGE is bilateral, synchronous, symmetric, and generalized spike-wave complex. Other classic EEG abnormalities are polyspikes, epileptiform K-complexes and sleep spindles, polyspike-wave discharges, occipital intermittent rhythmic delta activity, eye-closure sensitivity, fixation-off sensitivity, and photoparoxysmal response. However, admixed with typical changes, atypical epileptiform discharges are also commonly seen in GGE. There are circadian variations of generalized epileptiform discharges. Sleep, sleep deprivation, hyperventilation, intermittent photic stimulation, eye closure, and fixation-off are often used as activation techniques to increase the diagnostic yield of EEG recordings. Reflex seizure-related EEG abnormalities can be elicited by the use of triggers such as cognitive tasks and pattern stimulation during the EEG recording in selected patients. Distinct electrographic abnormalities to help classification can be identified among different electroclinical syndromes.

## Introduction

Genetic generalized epilepsy (GGE) encompasses several electroclinical syndromes diagnosed and classified according to clinical features and electroencephalographic (EEG) characteristics (1–3). The EEG hallmark of GGE is bilateral synchronous, symmetrical, and generalized spike-wave (GSW) discharges. Polyspikes and polyspike-wave discharges are also commonly seen in GGE. Fixation-off sensitivity (FOS), eye-closure sensitivity, photoparoxysmal response (PPR), epileptiform K-complexes/sleep spindles, and occipital intermittent rhythmic delta activity (OIRDA) are among the spectrum of abnormalities described in GGE (4).

In this review, we will be discussing the ictal and the interictal EEG abnormalities in GGE. We will also focus on the electrographic differences among different GGE syndromes, factors affecting the yield of EEG, and diagnostic pitfalls.

## Interictal Versus Ictal Abnormalities

Interictal EEG abnormalities are defined as “epileptiform patterns occurring singly or in bursts lasting at most a few seconds,” whereas ictal rhythms consist of “repetitive EEG discharges with a relatively abrupt onset and termination and characteristic pattern of evolution lasting at least several seconds” (5). Subclinical seizure activity refers to EEG seizure patterns not accompanied by clinical signs and symptoms (5). However, in absence seizures, differentiating interictal from ictal epileptiform discharges can be difficult as those discharges demonstrate monomorphic rhythmicity with little evolution. Consequently, the distinction between ictal and interictal activity depends on how long it lasts and clinical features, particularly impairment of consciousness during the discharge. Researchers have used several testing methods, including reaction time and motor tasks to study cognition and the degree of consciousness during spike-wave discharges (6).

Consequently, there is no consensus on the duration of the GSW paroxysm that defines an absence seizure. Sadleir et al. diagnosed absence seizures based on two criteria: (1) GSW activity of any duration when accompanied by clinical signs and (2) GSW lasting >2 s even if not accompanied by clinical correlates. Discharges of <2-s duration without clinical signs were identified as interictal fragments (7). A more recent study considered GSW bursts lasting 3 or more seconds, with or without clinical signs, as an absence seizure (8).

Conversely, myoclonic seizures and generalized tonic-clonic seizures demonstrate well-characterized EEG changes and the distinction from interictal EEG abnormalities is more unequivocal (4).

## Interictal Abnormalities

### Spike-Wave Complex

#### Morphology and Amplitude

**Figure 1:**
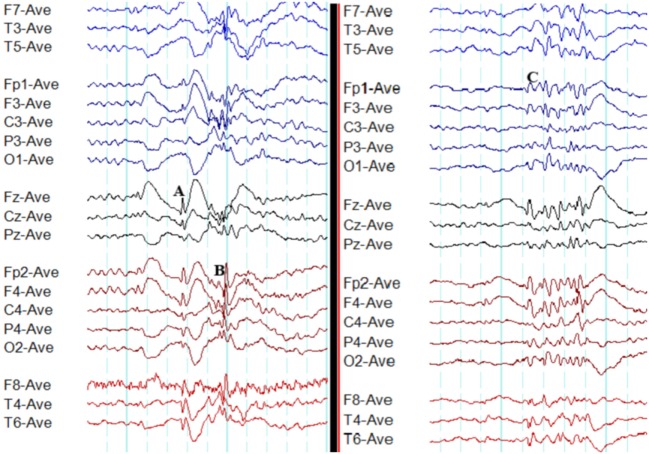
Typical interictal epileptiform discharges in genetic generalized epilepsy. Note bilateral, symmetrical, and synchronous spike-wave discharges **(A)**, polyspike-wave discharges **(B)**, and polyspikes **(C)**.

Gibbs et al. published the first detailed analysis of the spike-wave complex (9, 10). Subsequently, a more detailed analysis has revealed 3 components of the spike (spike 1, positive transient, and spike 2) (11). The surface negative spike 1 is of low amplitude (25–50 µV) and brief duration (10 ms). The second component is a positive transient of 100–150 ms. It is followed by spike 2 of negative polarity lasting 30–60 ms with frontal amplitude maxima. The dome-shaped wave of negative polarity, which follows the spike, lasts 150–200 ms (Figure 1) (11). However, spike 1 is seen less consistently than spike 2 (12).

A recent study based on 24-h ambulatory EEGs found 96.4% of generalized epileptiform discharges to be symmetric. However, the typical morphology was observed in only 24% (13).

#### Topography

Typically, the maximum amplitude is seen over the frontocentral region. With the use of 3-dimensional field potential maps, researchers were able to demonstrate that the amplitude maximum of spikes was over the frontal region involving anterior and midline electrodes (14). Using quantitative EEG analysis, Clemens and co-workers were able to demonstrate increased activity over the prefrontal region in patients diagnosed with GGE (15). The field maxima during absence seizures are usually detected at Fz electrode with lateral spread to F3, F4, and posterior spread to Cz electrode (16). The amplitude maximum of the spike-wave complex is most frequently observed in the frontocentral region (96.3%), followed by frontopolar (2.4%), and occipital (1.3%) regions (13).

Further insights into topography have been revealed in studies using quantitative EEG techniques. The source localization of epileptiform discharges on dense array EEG in juvenile myoclonic epilepsy (JME) detected activity in the orbitofrontal and medial frontopolar cortex (17). Another study using three techniques of source imaging analysis found anterior cingulate cortex and medial frontal gyrus as the primary anatomical sources of GSW discharges in GGE (18).

#### Regularity

In EEG, regularity is defined as “waves or complexes of approximately constant period and relatively uniform appearance” (5). The classic electrographic feature in GGE is regular and rhythmic GSW discharges. Nonetheless, a recent study has reported that 60% of GSW paroxysms are irregular (13).

#### Frequency of Discharges

The typical 3 Hz spike-wave activity characteristic of absence seizures was first described by Gibbs and collaborators (9). The fast spike-wave activity of >3.5 Hz is usually seen in juvenile myoclonic epilepsy (JME) (19). The spike-wave discharge frequency in juvenile absence epilepsy (JAE) (mean 3.25 Hz) is faster than childhood absence epilepsy (CAE) and slower than JME (7). In spike-wave paroxysms, the frequency is not constant throughout. The initial frequency is slightly faster and then it becomes more stable, slower, and regular (20).

#### Background

In GGE, typically, epileptiform discharges emerge from a normal background (2). Generalized epileptiform discharges occurring on a slow and disorganized background raise the possibility of an epileptic encephalopathy (21, 22).

### Polyspikes and Polyspike-Wave Discharges

Polyspikes are characterized by a run of two or more spikes, whereas the polyspike-wave complex consists of polyspikes followed by slow waves (5). In GGE, polyspikes usually occur in the form of high-amplitude rhythmic bursts with synchronized and generalized distribution (Figure 1).

### Photoparoxysmal Response

This is an abnormal response manifesting with the generation of spike-wave complexes, polyspikes, or polyspike-wave discharges during intermittent photic stimulation (5). The PPR is under the influence of several confounding variables including age, sex, ethnicity, genetics, antiepileptic medication use, state of alertness (sleep vs wakefulness), sleep deprivation, and the stimulation technique. There are three grades of PPR: (1) posterior stimulus dependent response, (2) posterior stimulus independent response, and (3) generalized response (23). The response to photic stimulation is defined as self-sustained when the epileptiform discharges outlast the stimulus by ≥100 ms (24). It is most frequently detected in JME (83%) followed by CAE (21%) and JAE (25%) (7). However, PPR can also be elicited in 0.3–4% adults without a history of epilepsy (25, 26). It is detected more frequently (14.2%) in asymptomatic children (27). The influence of various confounders including stimulation techniques may explain the wide range of results reported in the literature.

### Eye-Closure Sensitivity

**Figure 2:**
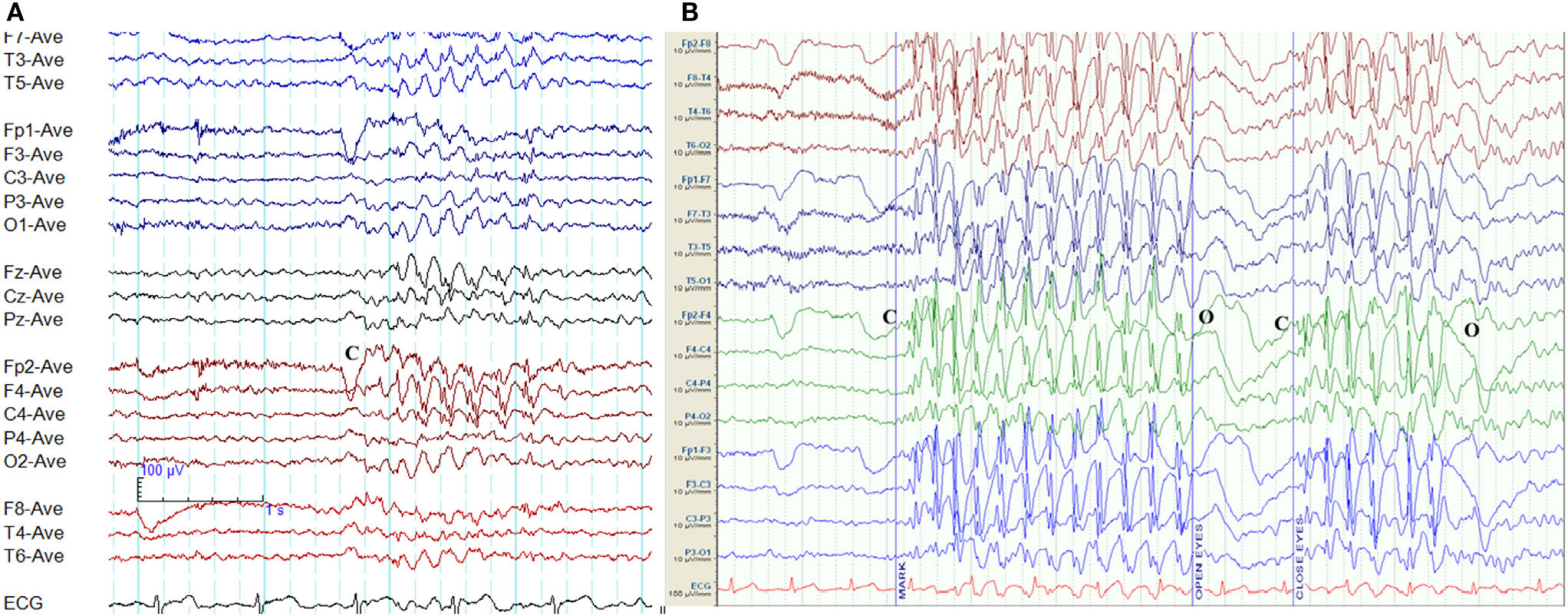
Eye-closure sensitivity and fixation-off sensitivity (FOS) in genetic generalized epilepsy. **(A)** Generalized spike-wave and polyspike-wave discharges appear after eye closure (C) and fades away after one second indicating eye-closure sensitivity. **(B)** Generalized epileptiform discharges appear with eye closure (C), continues as long as eyes are closed, and disappears on eye opening (O) indicating FOS.

Epileptiform discharges characteristic of eye-closure sensitivity emerge within 1–3 s of eye closure and last for 1–4 s. However, the discharges do not persist for the total duration when eyes remain closed (Figure 2). Photosensitivity and eye-closure sensitivity are related phenomena (28).

### Fixation-Off Sensitivity

Epileptiform discharges, generalized or occipital, triggered by the elimination of fixation and central vision are the hallmarks of FOS (29). This abnormality needs to be distinguished from photosensitivity and eye-closure sensitivity. In FOS, epileptiform discharges persist for the total duration of eye closure and disappear on eye opening (Figure 2) (29). To confirm FOS, central vision and fixation should be abolished with the application of spherical lenses, Frenzel lenses, or Ganzfeld stimulation technique (30). FOS has been described in GGE and occipital epilepsy (30). In some patients, photosensitivity and FOS coexist (31).

### Epileptiform K-Complexes and Sleep Spindles

**Figure 3:**
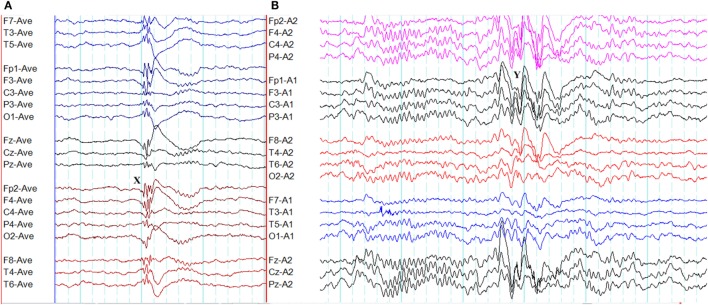
Epileptiform K-complexes and sleep spindles in genetic generalized epilepsy. **(A)** Polyspikes overlap with a K-complex at *X*. **(B)** A burst of generalized spike-wave discharges (*Y*) in the midst of a sleep spindle.

The overlap between generalized epileptiform discharges and K-complexes (epileptiform K-complexes) as well as sleep spindles (epileptiform sleep spindles) has been known to researchers for many decades (32, 33). This overlap generates complexes with a very characteristic morphology and topography (Figure 3) (33). A recent study has found this to be common in GGE with 65% of patients demonstrating epileptiform K-complexes and 10% epileptiform sleep spindles (34). These abnormalities probably indicate the link between microarousals and epileptiform discharges in GGE (34).

### Occipital Intermittent Rhythmic Delta Activity

Occipital intermittent rhythmic delta activity is characterized by transient unilateral or bilateral occipital runs of 2–3 Hz, regular, rhythmic, and sinusoidal delta activity (5). Deep stages of sleep, as well as eye opening, typically attenuate OIRDA, whereas drowsiness and hyperventilation make it more prominent (35). It is detected in approximately one-third of patients diagnosed with CAE (36). Though often reported as an EEG abnormality of CAE, OIRDA is not specific to epilepsy. It is occasionally seen in encephalopathies, particularly in children (35).

## Ictal EEG Changes

### Myoclonic Seizures

**Figure 4:**
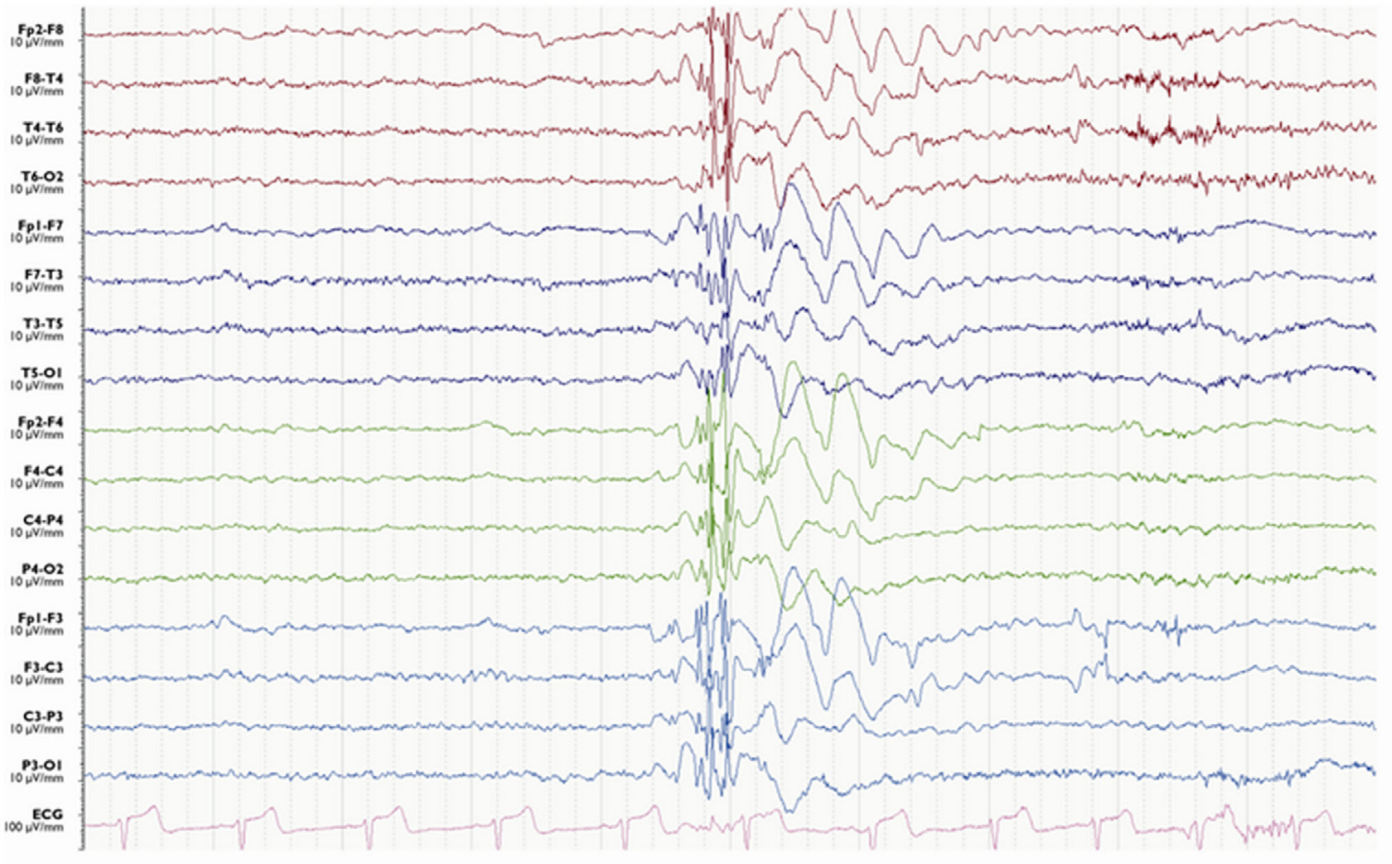
Electroencephalography of a myoclonic seizure. A burst of generalized polyspike-wave activity is followed a few slow waves.

High-amplitude, generalized, polyspike activity of 10–16 Hz with the frontocentral maximum is the EEG hallmark of myoclonic seizures (19, 37). These typical discharges may be preceded by irregular 2–5 Hz GSW activity and sometimes followed by irregular slow waves of 1–3 Hz (Figure 4) (19, 37, 38). The EEG seizure may be several seconds longer than the clinical seizure (37, 38).

### Typical Absence Seizures

**Figure 5:**
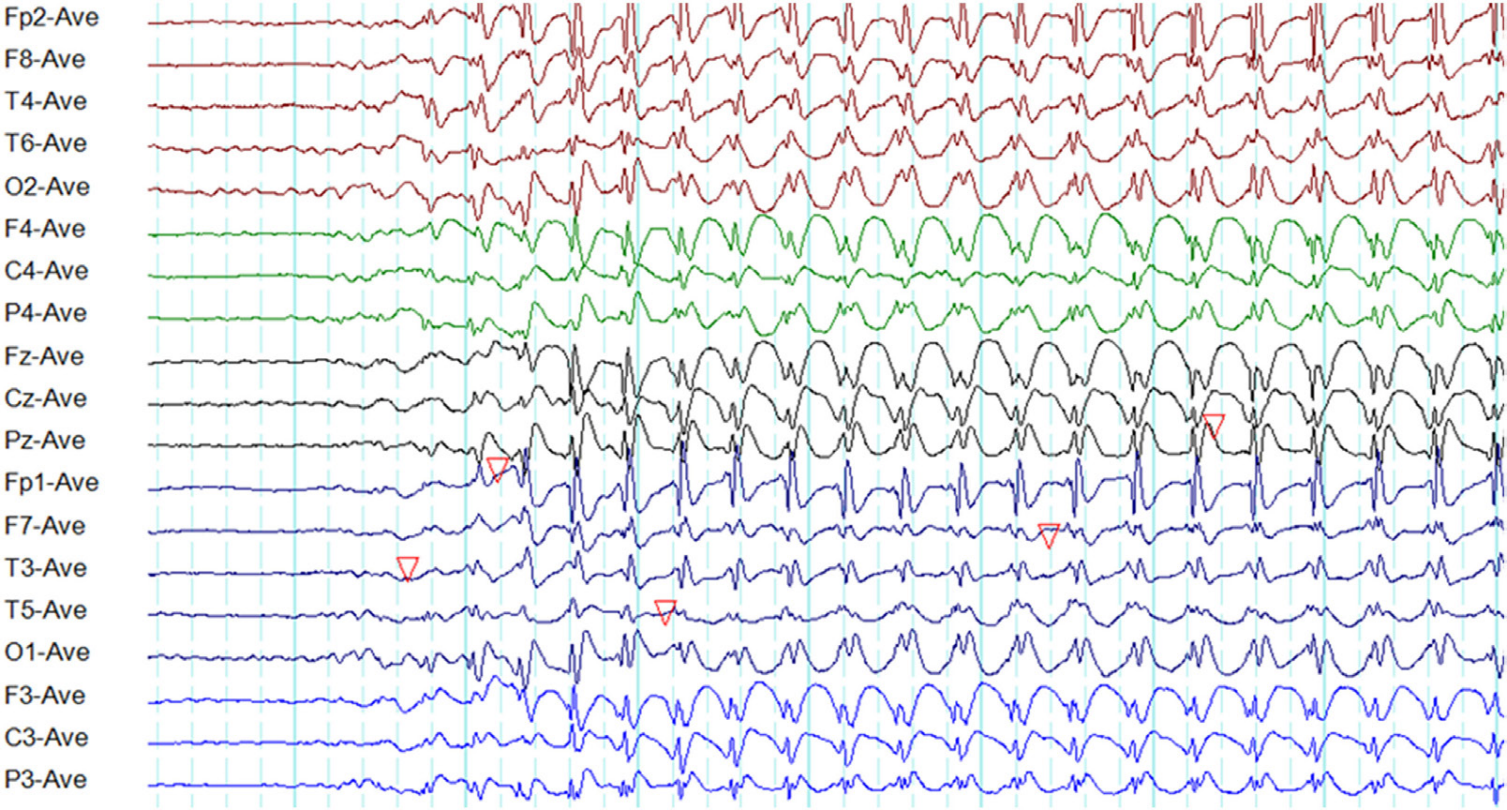
Electroencephalography of a typical absence seizure. Note the paroxysm of generalized, symmetrical, synchronous, and regular 3-Hz spike-wave discharges of frontocentral maxima.

Bilateral, regular, symmetrical, and synchronous 3-Hz spike-wave activity (range 2.5–4 Hz) sometimes admixed with polyspike-wave discharges on a normal background is the hallmark of a typical absence seizure (Figure 5) (39, 40). There are some differences among syndromes.

It is not unusual for the initial ictal discharge to be atypical. It could be non-generalized, spike-wave, polyspike-wave, or irregular discharges, with typical GSW activity appearing after an average of 0.7 s (7).

### Myoclonic Absence Seizures

**Figure 6:**
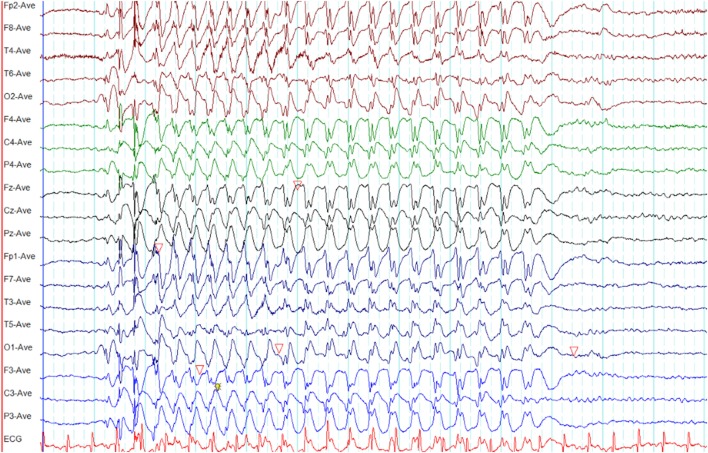
Electroencephalography (EEG) of a myoclonic absence seizure. Note the paroxysm of spike-wave discharges is similar to a typical absence seizure as illustrated in Figure 4 (time-base of this EEG = 20 s/page).

Myoclonic absence seizures are semiologically characterized by absences in association with tonic contractions resulting in progressive upper limb elevation and superimposed rhythmic myoclonic jerks (2). The impairment of awareness is less pronounced in comparison to typical absence seizures. Both myoclonic absence and typical absence seizure have similar ictal EEG patterns (Figure 6) (2, 41). Polygraphic recordings are used to demonstrate the correlation between the ictal EEG and tonic as well as myoclonic activity. Often triggered by hyperventilation, myoclonic absence seizures are less frequently (14%) induced by intermittent photic stimulation (41).

### Absence Seizures with Eyelid Myoclonia

**Figure 7:**
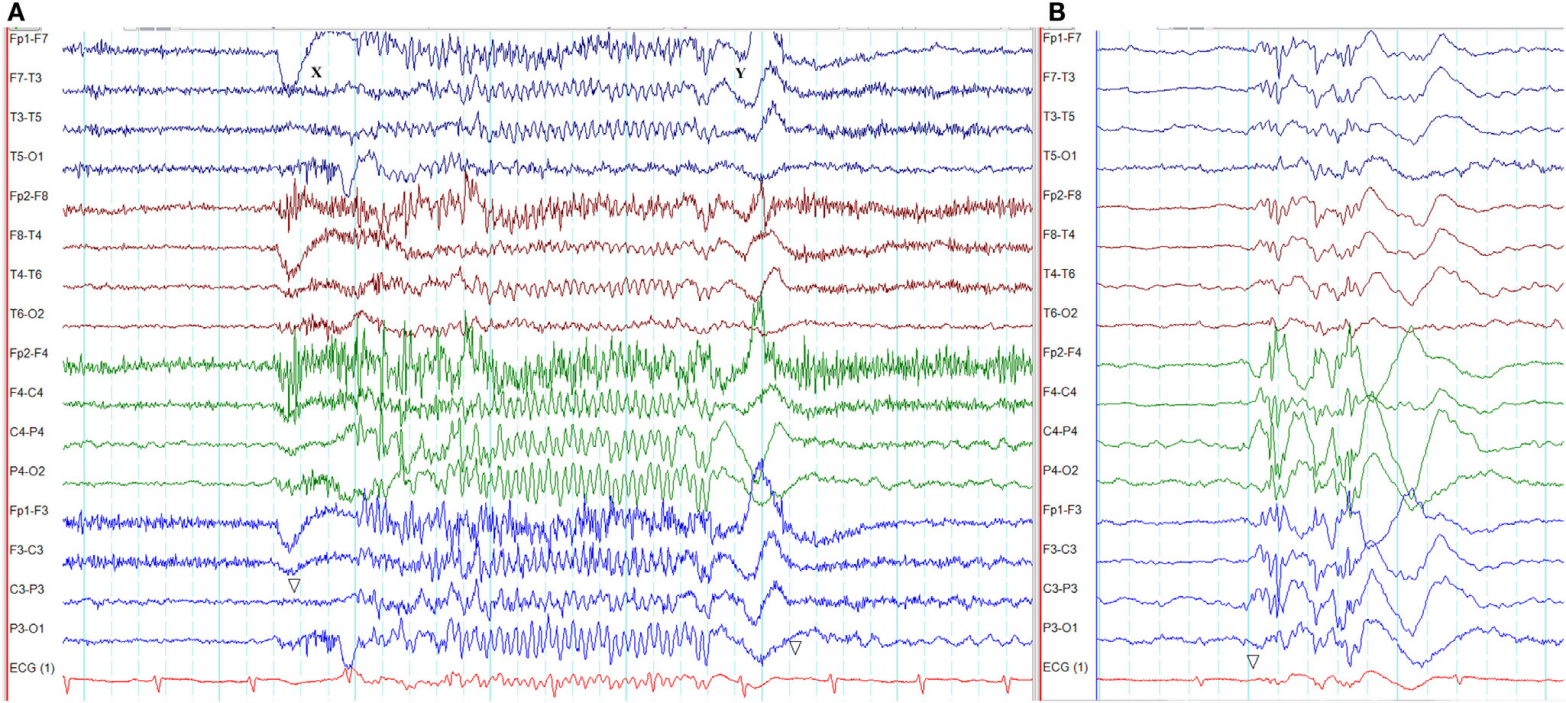
Electroencephalography of an absence seizure with eyelid myoclonia in Jeavons syndrome. **(A)** The absence seizure was triggered by eye closure at *X*. Note the paroxysm of generalized, fast polyspike activity (*X*–*Y*). This seizure was semiologically characterized by eyelid myoclonus, hyperextension of the neck, and unresponsiveness. **(B)** Interictal generalized polyspike-wave discharges during sleep recorded from the same patient.

Eyelid myoclonia with absences, EEG paroxysms/seizures triggered by eye closure, and photosensitivity are the main features of Jeavons syndrome (42). Sometimes, eyelid myoclonia may not be associated with an absence seizure (42). The ictal EEG typically shows generalized high-amplitude polyspikes and polyspike-wave discharges of 3–6 Hz lasting 1.5–6 s (Figure 7) (42). The ictal discharges occur with or before the onset of eyelid myoclonia (42). The EEG abnormalities are usually triggered by eye closure, intermittent photic stimulation, and hyperventilation (42). FOS may coexist (42).

### Generalized Tonic-Clonic Seizures

Muscle and movement artifacts mask the EEG during GTCS unless muscle relaxants are used to paralyze the subject. Generalized polyspike-wave bursts usually mark the ictal onset. Generalized amplitude attenuation follows, with or without low voltage, generalized, 20–40 Hz fast activity superimposing for a few seconds. The onset of the tonic phase coincides with the voltage attenuation. Then, generalized rhythmic alpha activity (10–12 Hz) evolves with increasing amplitude and decreasing frequency accompanied by the ongoing tonic phase. When the decreasing frequency reaches 4 Hz, repetitive polyspike-wave complexes emerge accompanied by myoclonic and clonic jerking semiologically. With the progression of the seizure, periodic bursts of polyspike-wave discharges appear with background suppression in between. Generalized EEG suppression is seen for a variable period with the termination of clonic jerking. The gradual recovery is marked by the restoration of the background rhythm from irregular generalized delta slowing proceeding to theta, and finally alpha rhythm (38).

## Atypical EEG Abnormalities

The typical EEG abnormalities in GGE are generalized, symmetrical, and bisynchronous epileptiform discharges. However, for many decades, atypical EEG abnormalities such as focal discharges, lateralized discharges, asymmetries, and irregular discharges have been reported in the literature (43–47). In absence seizures, the initial discharge has been found to be non-generalized in 50% (36).

**Figure 8:**
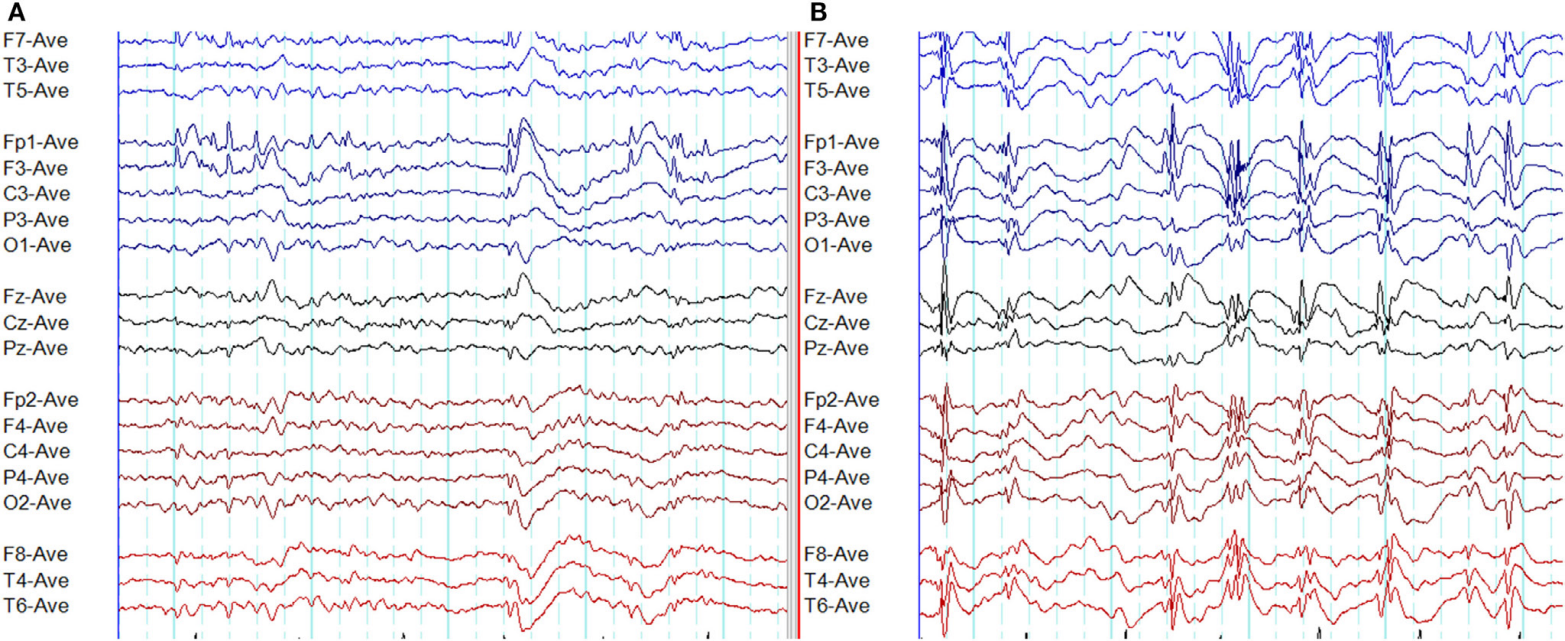
Atypical epileptiform discharges: amplitude asymmetry. **(A)** Note asymmetric epileptiform discharges with higher amplitude in the left frontal region. Synchronous epileptiform discharges of low amplitude are evident on the right on careful inspection. **(B)** More symmetric generalized epileptiform discharges recorded from the same patient.

**Figure 9:**
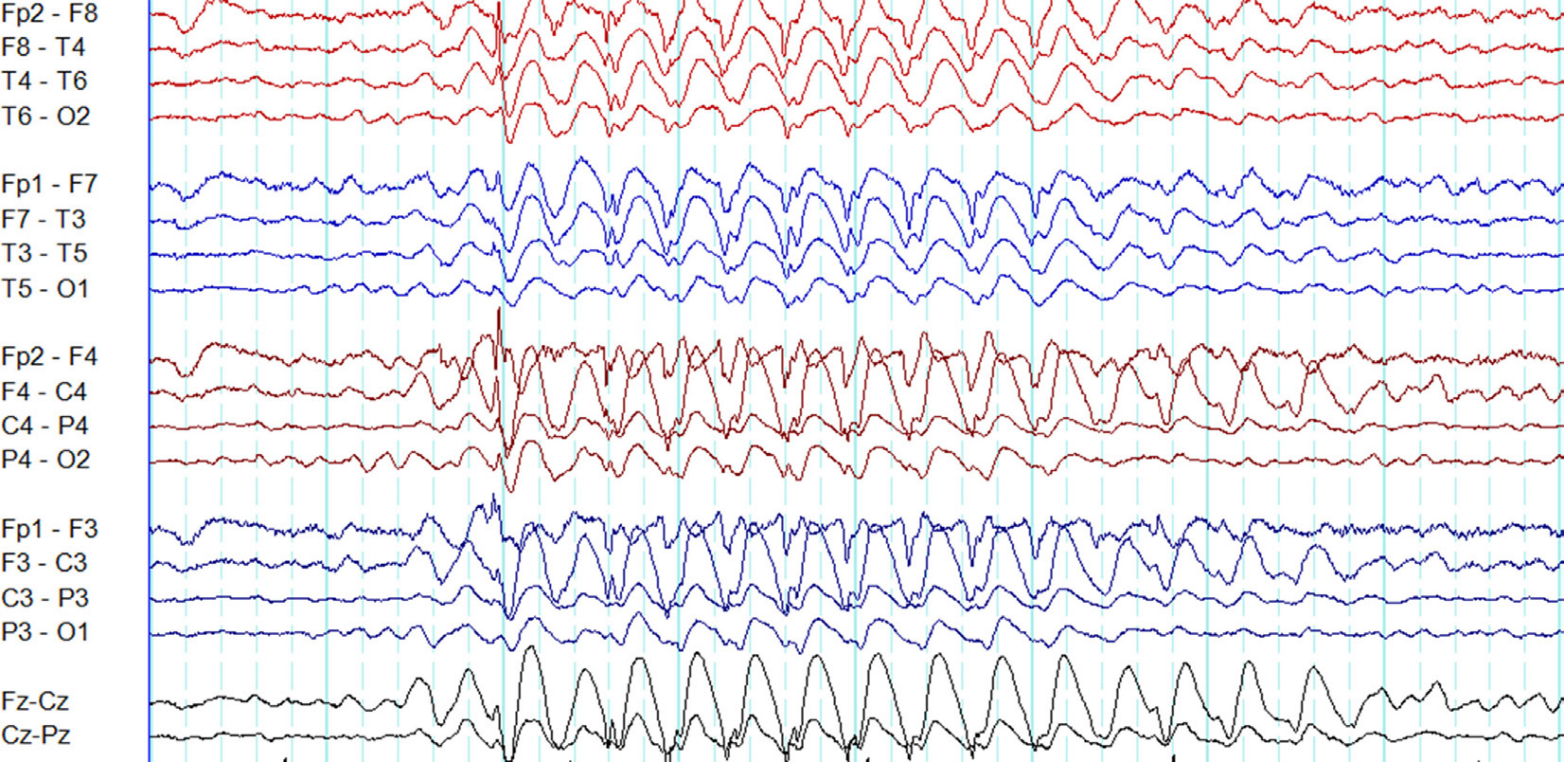
Atypical epileptiform discharges: focal onset and offset of paroxysms. A generalized spike-wave paroxysm in juvenile absence epilepsy. Note the focal onset and offset in the left frontal region.

**Figure 10:**
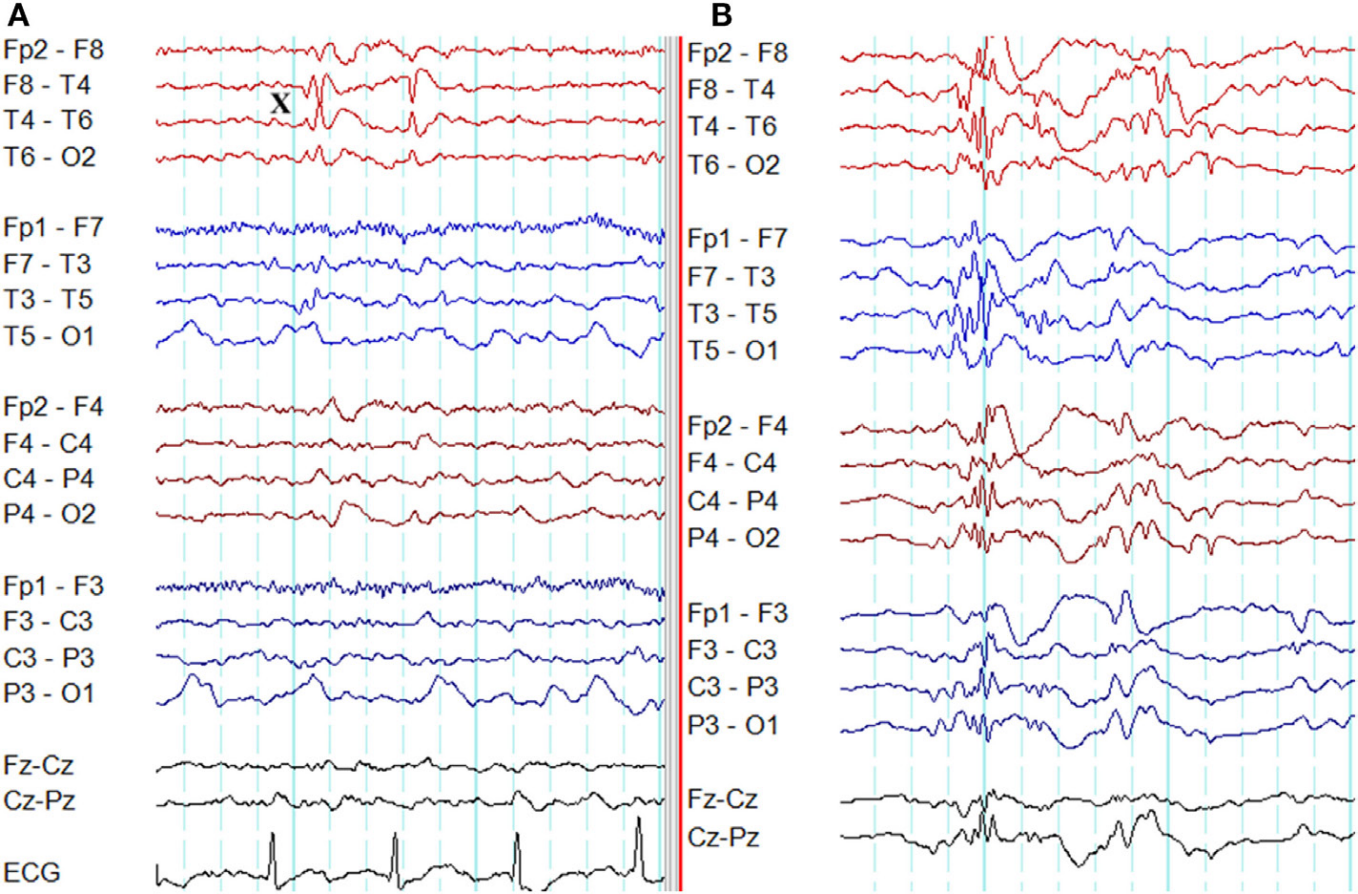
Atypical epileptiform discharges: focal discharges. **(A)** Note focal discharges at right temporal region (*X*). **(B)** Generalized epileptiform discharges recorded from the same patient.

**Figure 11:**
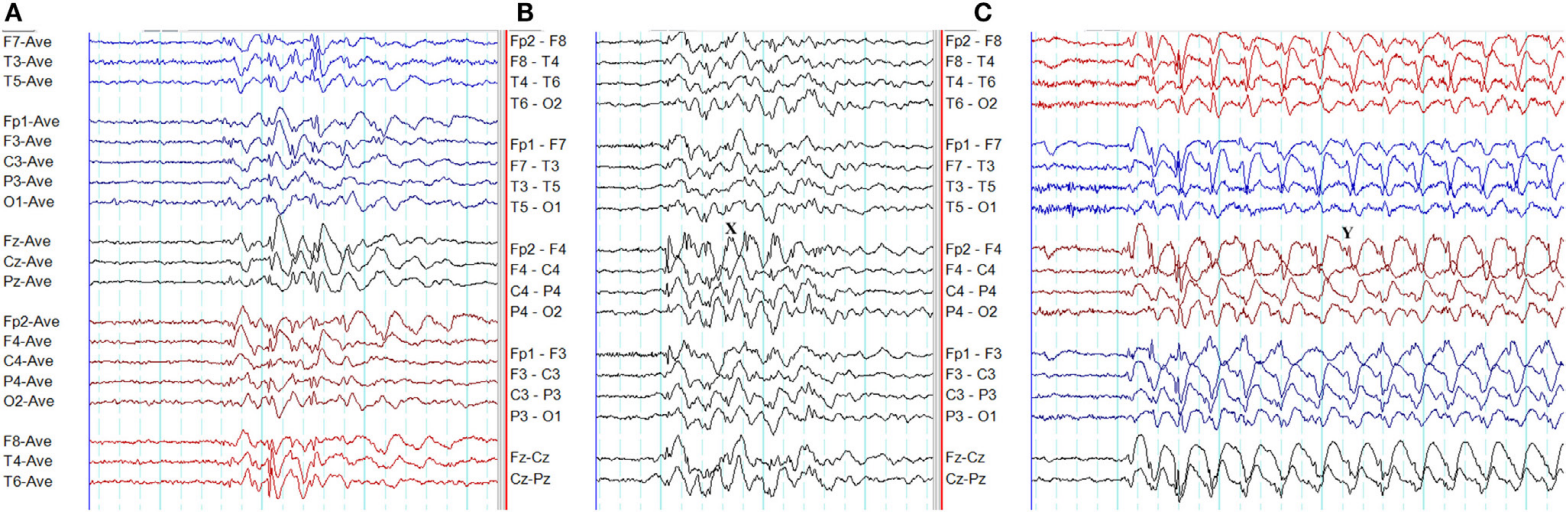
Atypical epileptiform discharges: abnormal morphology. **(A)** Waves without spikes. Note at the end of spike-wave paroxysms there are waves without preceding spikes. **(B)** Spikes overriding the waves. Note spikes on top of the wave at *X*. **(C)** Spikes overriding the waves. Note spikes on the descending limb of the preceding wave (*Y*).

**Figure 12:**
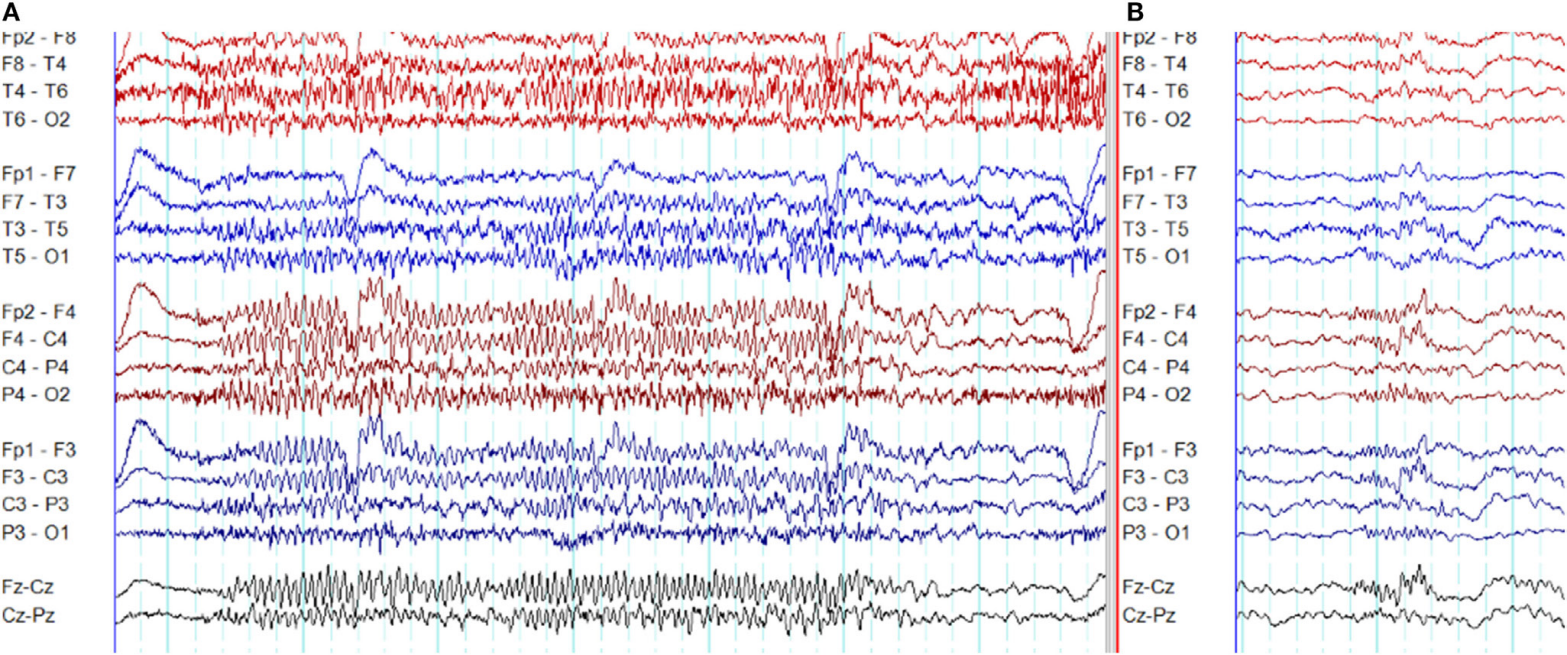
Atypical epileptiform discharges: generalized paroxysmal fast rhythm. **(A)** A run of generalized fast activity in wakefulness. **(B)** Similar changes during sleep.

A recent study based on 24-h ambulatory EEGs has quantified the atypical epileptiform EEG abnormalities in GGE (48). This study identified six atypical EEG abnormalities: (1) amplitude asymmetry, (2) focal onset of paroxysms, (3) focal offset of paroxysms, (4) focal epileptiform discharges, (5) abnormal morphology, and (6) generalized paroxysmal fast rhythm (Figures 8–12). It was found that 66% of GGE patients had at least one type of atypical abnormality in the 24-h EEG recording. Patients diagnosed with JAE and JME had those abnormalities most frequently, followed by epilepsy with generalized tonic-clonic seizures alone (GTCSA) and CAE. The most frequent atypical abnormality in the cohort was atypical morphology in 93.4% of patients. Other atypical EEG abnormalities were amplitude asymmetry (28%), focal discharges (21.5%), focal onset (13.1%), focal offset (8.2%), and generalized paroxysmal fast rhythm (1.9%) (48). It is of practical relevance to note that atypical abnormalities may result in misdiagnosis and delayed diagnosis (47).

## Provoking Factors Affecting the EEG

### Arousals, Sleep, Sleep Deprivation, and Circadian Rhythmicity

There are circadian variations in seizures and epileptiform discharges in GGE. Generalized spike-wave (GSW) activity is seen more often in non-rapid eye movement (NREM) sleep, but rare in rapid eye movement sleep (49). In GGE, sleep deprivation significantly increases the density of spike-wave discharges in both sleep and wakefulness (50). In JME, routine EEGs (without sleep deprivation) done in the morning are more often abnormal than those done in the afternoon (51). In JME, sleep EEG always shows epileptiform discharges (52).

Epileptiform discharges in GGE appear to be closely related to sleep–wake cycle. A retrospective study based on 24-h ambulatory EEGs found that 4.6% of patients had epileptiform discharges correlating with awakening. All patients who had epileptiform discharges on awakening were diagnosed with GGE. The epileptiform discharges were detected between 20 and 50 min following awakening in JME (53).

The interaction between circadian rhythmicity and the sleep–wake cycle in the generation of epileptiform discharges in GGE has been evaluated in a recent study (54). Epileptiform discharges are significantly shorter in duration and more frequent during the NREM sleep compared with wakefulness. When quantified, 67% of epileptiform discharges are detected in NREM sleep whereas 33% occurs in wakefulness. The distribution of epileptiform discharges demonstrates two peaks (11 p.m. to 7 a.m. and 12 noon to 4 p.m.) and two troughs (6 p.m. to 8 p.m. and 9 a.m. to 11 a.m.) (54). These findings highlight the variability in the diagnostic yield in relation to the time-of-day and sleep–wake cycle. The best time for the optimal yield of EEG abnormalities is from 11 p.m. to 7 a.m. Similarly, capturing natural sleep during the EEG recording significantly increases the diagnostic yield (54). Hence, 24-h ambulatory EEG is a very useful diagnostic tool in GGE.

### Hyperventilation

Hyperventilation is routinely used as an activation method in EEG. Hyperventilation-induced EEG abnormalities seem to depend on the severity of hypocapnia and the reduction in cerebral blood flow (55).

Hyperventilation often induces ictal and interictal abnormalities in children diagnosed with absence seizures (56). Hyperventilation triggered absence seizures in 67% of patients in a pediatric cohort (mean age 9.3 years) diagnosed with JAE and CAE (55). In untreated children, hyperventilation induces absence seizures more often in CAE and JAE (87% each) in comparison to JME (33%) (7). In contrast, another study involving a predominantly adult cohort, during hyperventilation, no one with generalized epilepsy had seizures and only 12.2% had an increase in interictal epileptiform discharges (57). Hyperventilation-induced GSW paroxysms were found in only 12.3% of adult patients with GGE on treatment (13). These studies suggest that during hyperventilation absence seizures are more likely to occur in the younger age group with untreated CAE and JAE.

### Photic Stimulation

Intermittent photic stimulation is a routine induction technique during EEG recordings. The PPR is more often seen in generalized epilepsy than in focal epilepsy. It is under the influence of several variables including age, sex, antiepileptic drug therapy, level of arousal, sleep deprivation, and the stimulation technique (4).

### Reflex Triggers

Reflex seizures on exposure to specific stimuli are sometimes encountered in GGE. The use of such stimuli as activating procedures during the EEG recording in selected patients is an option to improve the yield of EEG abnormalities.

Reflex seizures involving visual stimulation have been reported in several epilepsy syndromes including GGE symptomatic generalized epilepsy, and occipital epilepsy (58). Flickering lights, patterns, video games, and television are among the common visual triggers. Both photosensitivity and pattern sensitivity are implicated in television and video game induced seizures. Around 90% of patients with electrographic pattern sensitivity also demonstrate PPR (59). 3D television and movies do not pose a higher risk of reflex seizures than 2D television and movies (60).

Non-verbal cognitive stimuli such as thinking and praxis may induce reflex seizures in GGE. In a study involving reflex epilepsy triggered by spatial tasks, card or board games, and calculation, 96% experienced generalized tonic-clonic seizures often preceded by myoclonic jerks, whereas 68% demonstrated generalized epileptiform discharges on EEG (61). Another study involving 480 patients found that neuropsychological tasks provoked epileptiform discharges in 38 patients and 36 of those patients were diagnosed with GGE (62). Mental arithmetic and decision-making may trigger “noogenic” (thinking-associated) seizures among susceptible individuals. Cognitive activity in conjunction with planned motor tasks usually with hands is implicated in praxis-induced seizures (63). Praxis-induced reflex seizures are particularly common in JME (45, 62). Reading, talking, and writing are examples of verbal cognitive stimuli that may trigger reflex seizures. Both generalized and focal epilepsies have been reported under this category (63).

## EEG Differences among Syndromes

### Interictal EEG Abnormalities in Electroclinical Syndromes of GGE

Several electroclinical syndromes such as CAE, JAE, JME, and epilepsy with generalized epilepsy with tonic-clonic seizures alone (GTCSA) have been described in GGE. In this review, we will focus on the four main syndromes: CAE, JAE, JME, and GTCSA. It should be noted that apart from the electroclinical syndrome, epileptiform abnormalities in GGE are under the influence of many variables including sex, age, the state of alertness, activation procedures, techniques of EEG recording, and antiepileptic drug therapy (4).

### Interictal EEG in CAE

Childhood absence epilepsy is typically seen in children and the EEG signature is “generalized, bisynchronous, and symmetrical 3-Hz spike-wave discharges emerging from a normal background” (2). Fragments of GSW discharges are seen in >90% of cases, predominantly in drowsiness and sleep (7). Interictal polyspikes usually occur in drowsiness and sleep (7). Polyspike-wave discharges were detected in 26% of patients in a different series (64). Among untreated children with CAE, only 21% demonstrate PPR, whereas hyperventilation-induced absence seizures are seen in 87% (7).

Occipital intermittent rhythmic delta activity is seen in 20–30% of CAE subjects (8, 36), and 40% of those have a notched appearance (36).

### Interictal EEG in JAE

The onset of JAE is in teenage years (12–17 years). Absence seizures are less frequent but myoclonus is more common in JAE compared with CAE. In comparison to CAE, generalized tonic-clonic seizures more frequently precede the onset absence seizures in JAE (2). Fragmented discharges and polyspikes are seen in all patients, mostly in drowsiness and sleep (7).

### Interictal EEG in JME

Patients with JME typically experience their first seizure at puberty (12–18 years). The typical semiologic feature is myoclonic seizures predominantly involving arms. Generalized tonic-clonic seizures occur more frequently than absences (2). Sleep deprivation and alcohol are potent seizure triggers. Seizures, particularly myoclonus, frequently occur after awakening from sleep (19).

The classic EEG abnormalities in JME are generalized polyspikes and polyspike-wave discharges (19, 38). The interictal EEG is characterized by 3–6 Hz spike and polyspike-wave discharges in an irregular mix (28). Focal EEG abnormalities are common (45). PPR is seen in the majority (7). Both eye-closure sensitivity and FOS have been reported in JME (45).

### Interictal EEG in GTCSA

This condition is characterized by GTCS occurring on awakening or at random times. The median age of onset is (18 years) significantly older than JME and JAE (65). The interictal EEG demonstrates generalized polyspikes, polyspike-waves, and spike-wave discharges similar to other GGE syndromes. The mean spike-wave frequency is 3.6 Hz. The density of epileptiform discharges is significantly lower than CAE, JAE, and JME (66).

## Characteristics of Absence Seizures in GGE Syndromes

### Frequency of GSW Discharges

In all GGE syndromes, the initial frequency of GSW activity is faster. In the next phase, the discharges become more regular and slower in frequency by 0.4–0.6 Hz. The frequency decreases again in the terminal phase of CAE and JAE (20). The highest median frequency of GSW during the first second of an absence seizure is in JME (3.5 Hz). It is marginally slower in JAE (3.25 Hz) and CAE (3 Hz) (7). In JME, the GSW activity often tends to be faster (>3.5 Hz) (19, 38, 67). A more recent study based on 24-h EEGs found median GSW frequencies of 3.3 (CAE), 3.1 (JAE), 3.8 (JME), and 3.5 (GTCSA). But the differences were not statistically significant (66).

### Epileptiform Discharge Morphology and Duration

Childhood absence epilepsy and JAE demonstrate similar morphologies of GSW discharges. Multiple spikes preceding or overlapping slow waves give rise to an appearance of compressed “W”s in absence seizures of JME (20). The polyspike-wave activity is seen more often in JME and JAE than CAE (7). CAE and JAE have longer EEG seizure durations than JME (20, 68). The longest EEG absence seizure is seen in JAE, whereas the shortest is in GTCSA (66).

### Organization of Discharges

**Figure 13:**
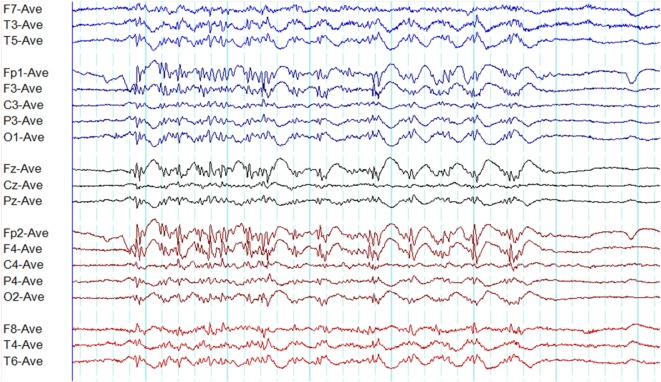
A disorganized (irregular) paroxysm of generalized epileptiform discharges in juvenile myoclonic epilepsy. Note this paroxysm has a mix of polyspikes and polyspike-wave discharges with varying frequency and morphology.

Absence seizures typically demonstrate well organized regular and rhythmic ictal EEG pattern. In disorganized discharges, regular rhythmic activity is interrupted by, (a) brief (<1 s) and transient interruptions in ictal rhythm, or (b) waveforms of different frequency and/or morphology (Figure 13) (7). Disorganized ictal discharges are 110 times more likely to occur in JME than CAE and eight times more likely in JAE than CAE (7). It is also influenced by provoking techniques, the state of arousal, and the age (7). Irregular and disorganized paroxysms are also seen in GTCSA though less frequently (66).

Table 1 summarizes key EEG differences among the four main GGE syndromes.

**Table 1:** Differences in electroencephalographic (EEG) features among syndromes.

	Reference	CAE	JAE	JME	GTCSA
GSWD frequency (Hz)	(66)	3.3	3.2	3.9	3.6
Irregular and disorganized paroxysms	(7)	Least common	8 times more likely than CAE	110 times more likely than CAE	NA
Percentage of GSWD fragments containing polyspikes	(7)	A—0, D—13%, S—40%	A—0, D—12%, S—24%	A—50%, D—50%, S—50%	NA
Photoparoxysmal response	(7)	21%	25%	83%	NA
Absence seizures during hyperventilation	(7)	87%	87%	33%	NA
Mean duration of paroxysms (s)	(66)	2.8	4.6	3.2	2.5
Total spike density	(66)	+	+++	+++	+
Density of generalized paroxysms	(66)	+	+++	+	+
Density of polyspikes and polyspike-wave discharges	(66)	+	+++	++	+
Density of pure GSWD	(66)	++	++	+	+

*CAE, childhood absence epilepsy; GSWD, generalized spike-wave discharges; GTCSA, generalized tonic-clonic seizures alone; JAE, juvenile absence epilepsy; JME, juvenile myoclonic epilepsy; NA, not available; A, awake; D, drowsy; S, sleep; density; duration of epileptiform discharges (in seconds) per an hour of EEG recording; +++, highest value; +, lowest value; +++, middle value; pure GSWD, fragments and paroxysms containing only spike-wave discharges (without any polyspikes or polyspike-wave discharges) (7, 66)*.

## Underpinning Network Mechanisms of GSW Complex

Currently, epilepsy is considered to be a disorder of network pathways. This concept is reflected in the current International League against Epilepsy terminology defining generalized seizures as those involving both cortical and subcortical bilateral networks (1). Hence, in GGE, the seizure activity originates at a certain point within the epileptic network and then rapidly engages bilaterally distributed network pathways (1).

Many animal and human experiments highlight the importance of frontal lobe and thalamus in the formation and propagation of GSW complexes. In the rat model, absence seizures originate from the somatosensory cortex rapidly spreading to the thalamus (69). In their pioneering work, Bancaud et al. recorded GSW discharges with the mesial frontal cortex stimulation (70). More recently, novel EEG techniques have provided intriguing insights into the underpinning epileptic network pathways in GGE.

### Dense Array EEG and Source Localization

A study based on dense array EEG in absence seizures has demonstrated spike-wave discharge onset in the dorsolateral frontal and orbital frontal regions followed by rapid and stereotypic propagation (71). Electrical source analysis of dense array EEG data has revealed frontotemporal networks involving the slow wave and spike propagation through ventromedial frontal networks during absence seizures (72).

### Magnetoencephalography (MEG)/EEG

A combined MEG/EEG study has described a prefrontal–insular–thalamic network in absence epilepsy (73). In JAE, the spike-wave discharge onset is in focal cortical regions with subsequent involvement of the default mode network as demonstrated by synchronous MEG/EEG data (74).

### Simultaneous EEG and Functional MRI (EEG-fMRI) Studies

Electroencephalographic and functional MRI is a non-invasive technique to measure regional brain activation during epileptiform discharges using blood oxygenation level-dependent contrast (75). A recent critical review has elicited three key features among EEG-fMRI findings in GGE: (a) activation of the thalamus, (b) activation of cortical regions, particularly frontal, and (c) deactivation of default mode areas (47).

### Combined Transcranial Magnetic Stimulation (TMS) and EEG Studies

Combined TMS and EEG is an emerging non-invasive technique with a potential to study the functional connectivity of the brain (76). A protocol to study GGE patients with TMS-EEG has been recently described (77). However, to date, changes in network connectivity have mostly been studied with TMS-EEG in focal epilepsy (78), while evaluation in GGE remaining in its infancy (79).

### Graph Theory and EEG

Graph theory is a mathematical concept to study brain connectivity. It describes networks in terms of interrelationship between nodes (brain regions) and edges (connections) (80). Graph theory is increasingly being used as a tool to analyze epileptic networks. A recent study has reported increased local connectivity in the frontal regions with spike-wave discharges in JME (81). Another study based on graph theory using EEG data found similarities in network topology between patient with GGE and their unaffected relatives (82).

Conclusions should be drawn with care from these studies due to various limitations. There is wide variability in the methodology among studies. In particular, EEG-fMRI studies vary in terms of study paradigms, and methods of data acquisition as well as analysis. Additionally, most studies are based on GSW activity. In GGE, there are other EEG abnormalities and underpinning network mechanisms may be different in those. Despite such limitations, there is growing support for the hypothesis that spike-wave discharges originate from a cortical focus with rapid spread to the thalamus followed by entrainment of the cortico-thalamo-cortical loop resulting in the classic GSW activity observed in GGE (83).

## Diagnostic Tools

Routine outpatient EEG, sleep-deprived outpatient EEG, short-term outpatient video-EEG, inpatient video-EEG, and 24-h ambulatory EEG are common tools used to diagnose and classify epilepsy in routine clinical practice. The yield is influenced by several variables such as age, AED therapy, pretest probability of epilepsy, provoking techniques used, the length of the recording, and the state of arousal (4).

The yield of interictal epileptiform discharges in the routine outpatient EEG is around 28% (84). After the first seizure, the average yield is 29% according to a systematic review (85). Serial EEGs appear to increase the diagnostic yield (86). One study based on outpatient short-term video-EEG found the yield to be 17.2% (87). However, in this study, 22% of patients had the test with the clinical diagnosis of psychogenic non-epileptic seizures reducing the yield of epileptiform discharges. Inpatient video-EEG monitoring has a higher yield (epileptic seizures 43.5%; interictal epileptiform discharges 43%) (88), but is an expensive test with limited availability.

Sleep EEG can be considered the most effective diagnostic tool as 67% of generalized epileptiform discharges occur in NREM sleep (54). Sleep deprivation appears to increase this yield further. Following sleep deprivation, GSW discharge densities increase in both sleep and wakefulness with the highest densities recorded in NREM sleep stages 1 and 2 (50). Though results in the literature are variable, sleep deprivation appears to increase the yield of epileptiform discharges (focal and generalized) by about 30% beyond the effect of sleep (89).

The use of multiple provoking techniques increases the diagnostic yield of EEG. A recent study reported a video-EEG protocol incorporating several provoking methods such as sleep deprivation, neuropsychological activation (language and praxis), hyperventilation, eye closure, intermittent photic stimulation, sleep, and arousal (90). The video-EEG was recorded for 4–6 h. Interictal epileptiform discharges were detected in 85.8% of patients, whereas 54.9% had seizures during the recording (90). The high yield might have been influenced by the fact that all patients in the cohort had an established diagnosis of GGE. Yet, this study demonstrates the importance of combining multiple provoking techniques to enhance the diagnostic yield.

Recent research indicates 24-ambulatory EEG to be a very useful test to diagnose and classify GGE (54). Its diagnostic sensitivity is 2.23 times higher than routine EEG (91). Ambulatory EEG recordings are very effective for several reasons. First, two-thirds of epileptiform discharges appear on sleep EEG recording and ambulatory EEG the most practical method to capture the natural sleep and increase the diagnostic yield (54). Second, epileptiform discharges in GGE demonstrates a time-of-day dependence with two peaks (11 p.m. to 7 a.m. and 12 noon to 4 p.m.) and two troughs (6 p.m. to 8 p.m. and 9 a.m. to 11 a.m.) (54). Routine outpatient EEG is likely to miss the most significant first peak (11 p.m. to 7 a.m.) while the 24-h ambulatory will capture both peaks. Third, it is four times cheaper than inpatient video-EEG (92). Finally, home-based ambulatory EEG is more convenient and acceptable to patients than hospital-based inpatient monitoring (93).

## Diagnostic Pitfalls

### Misdiagnosis of People without Epilepsy As Generalized Epilepsy

Paroxysmal disorders ranging from syncope to psychogenic non-epileptic seizures can be misdiagnosed as epilepsy. The rate of misdiagnosis is as high as 20–30% in general practice and outpatient clinics (94, 95). Misdiagnosis is likely to happen when an individual presenting with a non-epileptic disorder undergoes an EEG test yielding epileptiform abnormalities. It has been shown that 0.5% of healthy adults in the general population have epileptiform abnormalities in the EEG (25). Among healthy school children, the prevalence of GSW activity in the EEG is 0.9% (96). Epileptiform discharges are more frequently (37%) detected among the offspring of patients with epilepsy. 6% of healthy first-degree relatives of JME probands demonstrate typical generalized epileptiform discharges (97, 98). Hence, we wish to emphasize the importance of clinical correlation of EEG abnormalities in establishing the diagnosis of epilepsy.

### Misdiagnosis of Generalized Epilepsy As Focal Epilepsy

Atypical features, including focal epileptiform discharges, can potentially result in delayed diagnosis and misdiagnosis of GGE. The rate of misdiagnosis can be as high as 91% and the mean delay to the diagnosis ranges from 6 to 15 years in studies (47). As a result, many patients receive inappropriate antiepileptic drugs such as carbamazepine leading to paradoxical worsening of some seizures (47).

### Misdiagnosis of Focal Epilepsy As Generalized Epilepsy

#### Secondary Bilateral Synchrony

Tukel and Jasper coined the term “secondary bilateral synchrony” while reporting a series of patients with parasagittal lesions in whom the EEGs demonstrated bilaterally synchronous bursts of spike-wave complexes (99). Along with Penfield, they postulated that “a cortical focus can fire into subcortical structures and set off a projected secondary bilateral synchrony” (99). Subsequently, a stereo-EEG study reported that stimulation of the mesial frontal region induced paroxysms of bilaterally synchronous and symmetrical spike-wave discharges (70).

Blume and Pillay proposed three diagnostic criteria for secondary bilateral synchrony; (1) ≥2 s of lead-in time, (2) focal triggering spikes having a different morphology from the bisynchronous discharges, and (3) both triggering spikes and focal spikes from the same region having similar morphology (100). This is a rare phenomenon occurring in 0.5% of patients undergoing EEGs and is most frequently seen in association with frontal lobe foci (100).

#### Frontal Lobe Epilepsy: The Conundrum of “Pseudo Bilateral Synchrony”

**Figure 14:**
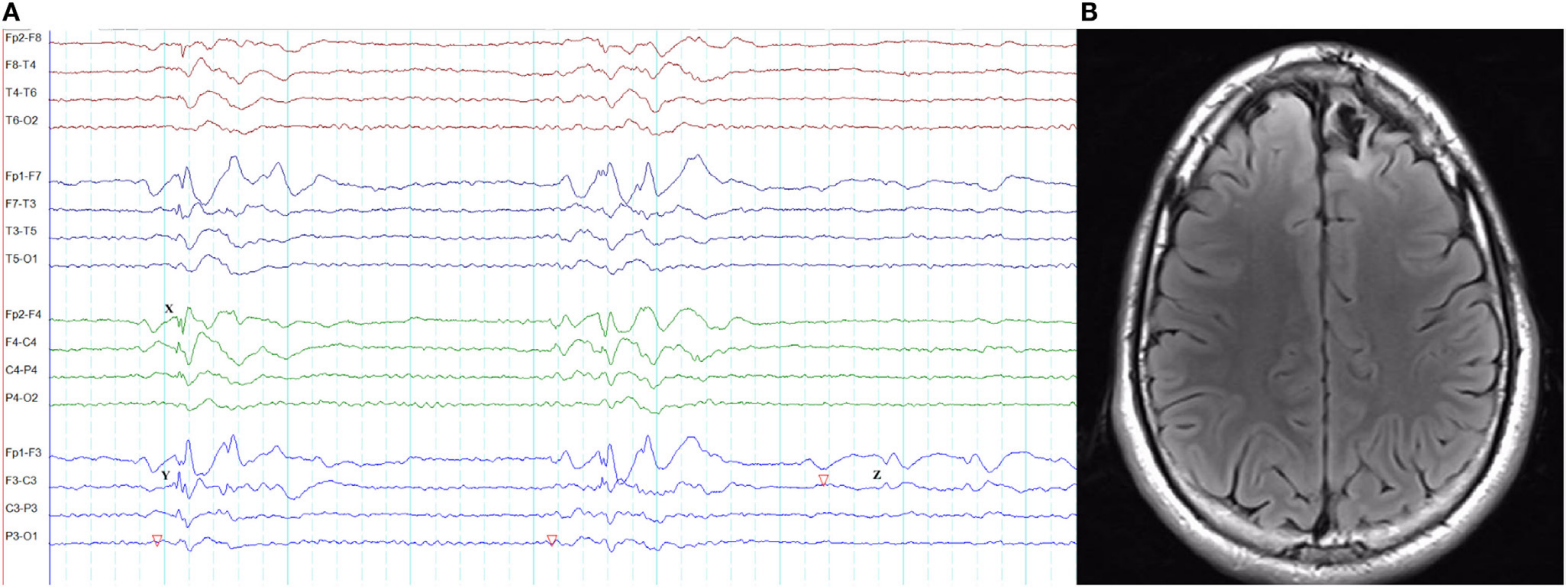
Pseudo bilateral synchrony in frontal lobe epilepsy. This patient presented with seizures following the surgery for left frontal brain abscess in the past. **(A)** In this longitudinal bipolar montage, bifrontal polyspike-wave discharges (*X, Y*) appear synchronous. However, focal sharp wave discharges are evident involving F3 electrode at *Z*. **(B)** The MRI demonstrating left frontal encephalomalacia.

**Figure 15:**
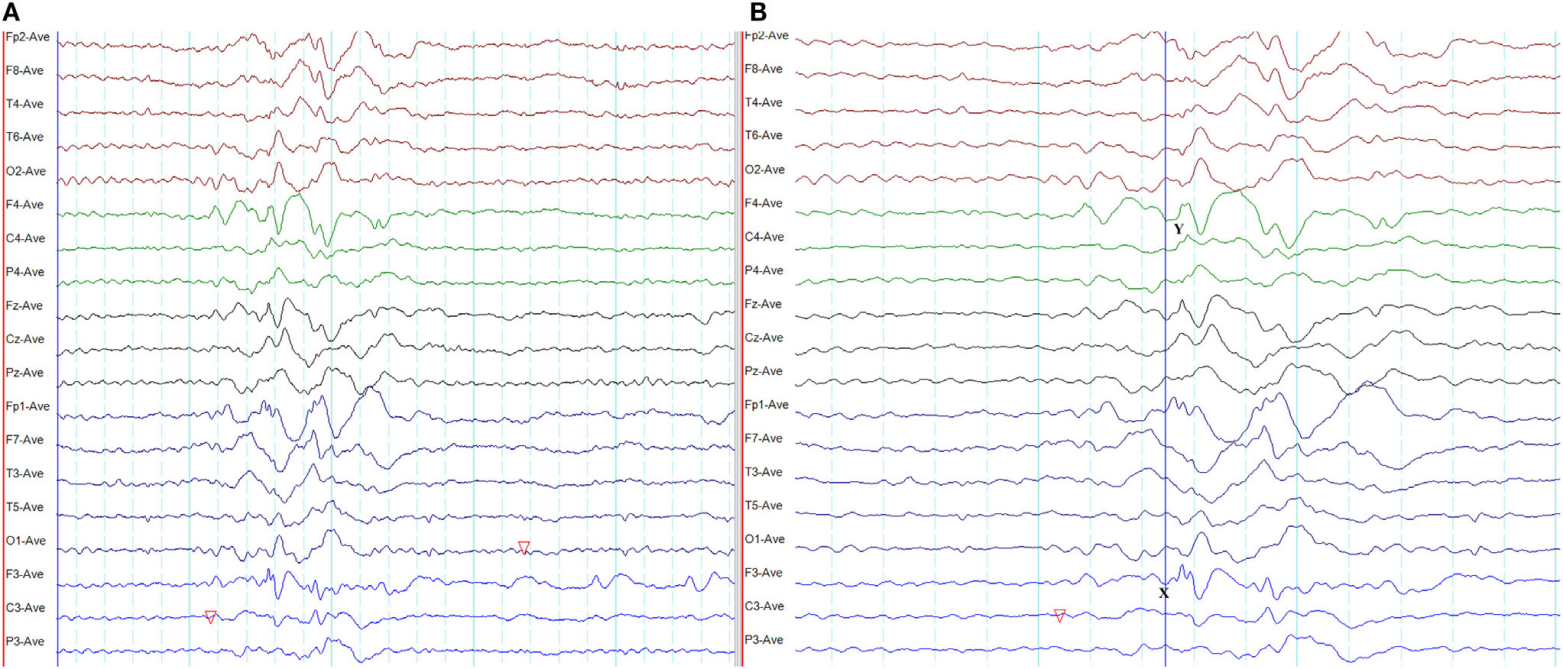
Pseudo bilateral synchrony in frontal lobe epilepsy. **(A)** This average referential montage demonstrates the same activity seen in (A) of Figure 14. The discharges appear bifrontal. Note focal discharges involving F3 and C3 (time-base of the electroencephalography = 10 s/page). **(B)** When the time-base is expanded to 5 s/page, it becomes clear that the epileptiform discharge emerges first on the left at Fp1 and F3 (*X*), followed by activity on the right (*Y*) confirming pseudo bilateral synchrony.

In frontal lobe epilepsy, the interictal epileptiform abnormalities range from focal to bilateral synchronous discharges. In a surgical series of frontal lobe epilepsy, 9% had bifrontal independent interictal epileptiform discharges whereas bilaterally synchronous discharges were recorded from 37% of patients (101). Epileptiform discharges recorded on the scalp EEG represent the summated activity of volume conduction and cortico-cortical propagation. Cortico-cortical propagation gives rise to asynchronous discharges with a time a lag. However, small time lags may not be appreciated by visual inspection and can be interpreted as synchronous discharges (102, 103). Hence, it is conceivable that frontal foci, particularly located in the midline, can generate bifrontal epileptiform discharges with “pseudo bilateral synchrony” that can be mistaken for truly bisynchronous discharges of GGE. Computer-aided analysis (104) or specific re-montaging (reference-subtraction montage) (103) can be used to detect the time lag between the electrodes and demonstrate that bilateral discharges are not truly synchronous but generated from a single a focus. Expanding the time-base of digital EEG is also a useful manipulation to detect time differences between seemingly synchronous discharges on two separate channels (Figures 14 and 15) (105).

### Misdiagnosis of Normal Variants As Generalized Epileptiform Discharges

The 6-Hz spike-wave (phantom spike-wave) pattern consists of bursts of generalized symmetric spike-wave discharges with a very low amplitude spike component (106). The bursts are typically very brief but can last up to 4 s on rare occasions (106). The amplitude maxima can be anterior or posterior (107). This variant, particularly the type with posterior maximum, usually emerges from drowsiness and disappears during deep sleep. Though the typical frequency is 6 Hz, it can range from 4 to 7.5 Hz (107). The spike component is <25µV in the majority and >75 µV in 5% (107). This is a benign variant of no clinical significance.

The 14- and 6-Hz positive burst pattern (14 and 6 Hz positive spikes or ctenoids) is a benign variant most often seen in early teens, which then becomes infrequent with advancing age. These spikes are surface positive in polarity, occurring in bursts of <1 s, with unilateral or bilateral distribution and posterior dominance during drowsiness and light sleep (106). This can be mistaken for polyspikes, but careful analysis of the polarity, frequency, and distribution should help clarify the diagnosis.

Small sharp spikes (benign sporadic sleep spikes) are seen in adults during drowsiness and light sleep. It disappears in deep sleep. The sharp waves are usually diphasic with a low amplitude (<50 µV) and brief duration (<50 ms) without an after-going slow wave. The spikes occur in the form of isolated transients with a unilateral or bilateral widespread field most prominent in the temporal regions (106).

## Gaps in the Literature and Future Directions

Drawing robust conclusions from the literature is challenging due to wide variability in methodology. Additionally, EEG abnormalities in GGE are influenced by numerous confounding variables affecting the results. Most studies are descriptive in nature limiting the option of drawing statistical conclusions. Finally, most studies are based on short-term EEG recordings.

To circumvent these shortcomings, prospective studies in drug naïve populations with GGE using standardized EEG recording protocols are needed. Longer (≥24 h) EEG monitoring is required to study both circadian and infradian rhythms. Additionally, there is a potential role for long-term EEG monitoring to evaluate seizure control and occupational safety including fitness to drive. The application of machine-learning technologies allows more rapid and accurate identification of abnormalities from large volumes of long-term recordings and permits accurate quantification that may provide new insights into classification, prognosis, and clinical outcomes.

More research should focus on the distinction and interrelationship between interictal and ictal epileptiform discharges. From the clinical perspective, more analytical studies are needed to delineate EEG differences among GGE syndromes. Beyond its routine clinical interpretation, EEG data can be used for computational modeling to study network dynamics (108). Research on network analysis in GGE needs to focus on all types of epileptiform abnormalities and associated networks.

## Conclusion

As highlighted in this review, there are several typical EEG features of GGE. The occurrence of atypical features, in particular, focal changes, should be borne in mind to avoid misdiagnosis. The use of provoking stimuli such as sleep deprivation, intermittent photic stimulation, hyperventilation, FOS, and reflex triggers during EEG recording can help increase the diagnostic yield. Some EEG features help differentiation among electroclinical syndromes. However, it should be emphasized that such differences are also influenced by several confounding variables including sex, age, state of alertness, activation methods, technical factors, and antiepileptic drug therapy.

## Ethics Statement

This study was conducted with approvals from the Human Research Ethics Committees of Monash Health and St. Vincent’s Hospital, Melbourne.

## Author Contributions

US: study concept and design, literature search, drafting, and critical revision of the manuscript. MC and WD: study concept, critical revision of the manuscript.

## Conflict of Interest Statement

The authors declare that the research was conducted in the absence of any commercial or financial relationships that could be construed as a potential conflict of interest.
